# “I have to do things differently now, but I make it work”—young childhood cancer survivors’ experiences of self-management in everyday living

**DOI:** 10.1007/s11764-021-01066-y

**Published:** 2021-06-07

**Authors:** Marie H. Larsen, Elna H. Larsen, Ellen Ruud, Anneli Mellblom, Sunniva Helland, Hanne Cathrine Lie

**Affiliations:** 1 Department of Behavioural Medicine, Institute of Basic Medical Sciences, Faculty of Medicine, University of Oslo (UIO), Postboks 1111, Blindern, 0317 Oslo, Norway; 2 Department of Paediatric Haematology and Oncology, Division of Paediatric and Adolescent Medicine, Oslo University Hospital, Rikshospitalet, Postbox 4950, Nydalen, 0424 Oslo, Norway; 3 Institute of Clinical Medicine, Faculty of Medicine, University of Oslo (UIO), Postboks 1111, Blindern, 0317 Oslo, Norway; 4 Regional Centre for Child and Adolescent Mental Health Eastern and Southern Norway (RBUP), Postboks 4623 Nydalen, 0405 Oslo, Norway; 5 The Children and Youth Clinic, Haukeland University Clinic, Jonas Lies vei 65, 5021 Bergen, Norway

**Keywords:** Cancer, Oncology, Childhood cancer survivors, Self-management, Qualitative interviews, Late effects

## Abstract

**Purpose:**

Living with late effects can affect young childhood cancer survivors’ (CCSs) self-management (SM) abilities. In this study, we explored different approaches to SM of everyday life by young CCS.

**Methods:**

This is a sub-study of a larger study on Physical Activity among Childhood Cancer Survivors (the PACCS study). We conducted individual interviews with 22 CCS aged 9 to 18 years who were at least 1 year off-treatment. An hybrid inductive-decductive thematic analysis was used.

**Results:**

Three main themes were identified: (1) managing everyday life with fatigue, (2) building self-management competence, and (3) cancer survivor as part of identity. Late effects, especially fatigue, contributed to a perceived ability gap compared to peers, limiting participation in everyday activities. CCS developed new SM skills to overcome such challenges and pushed themselves physically and mentally to master and balance activities and rest to regain energy. CCS changed activities, adapted their expectations, or legitimized their apparent lack of SM skills to regain a sense of self-efficacy. Managing the impact of cancer on relationships with family and friends also required use of SM strategies.

**Conclusions:**

The findings expand our currently limited knowledge of young CCS and SM skills they develop to manage everyday life after treatment completion. These, combined with ongoing support from family and peers, “make it work”.

**Implications for Cancer Survivors:**

The perspectives of young CCS illustrate their SM skills and support needs beyond transitioning off-treatment. Conceptualizing this within follow-up care may contribute to a feeling of mastery and increased satisfaction among CCS.

## Background

Treatment progress has resulted in dramatic increases in survival for patients diagnosed with childhood and adolescence cancers, yet receiving such a diagnosis remains a life-changing event [1]. Surviving cancer comes at a cost, with ongoing high risks of treatment-related late effects that can occur across survivors’ lifespan [2–4]. Late effects encompass health problems such as fatigue, heart failure, hormone failure, fertility problems, secondary cancer, and cognitive impairment [4–6]. Fatigue is described as one of the most common late effects, defined as “a distressing, persistent, subjective sense of physical, emotional, and/or cognitive tiredness or exhaustion related to cancer or cancer treatment, not proportional to recent activity and interferes with usual functioning” [7 , p. 9]. Each childhood cancer survivor (CCS) has a unique late effect risk profile, depending on genetic predisposition, diagnosis, age at diagnosing, treatment history, personal vulnerability, and resilience [4, 8, 9]. Many CCS attend medical follow-up for an extended period post-treatment to manage their health and screen for late effects [10].

Late effects such as fatigue, myocardial infection, neuropathic pain, cognitive dysfunction, and depression may become chronic medical health problems affecting physical and psychosocial functioning and quality of life, thereby requiring management strategies [11–14]. Given the persisting risk and chronicity of late effects, it has been advocated to consider cancer as a chronic disease and that patients with cancer should be cared for accordingly [15, 16]. Besides, the provision of self-management support in routine care for cancer survivors lags behind that of chronic diseases [17]. Similar to others with chronic conditions, cancer survivors must develop the ability to advocate for their health care, learn about their cancer disease and treatment, interpret the signs and symptoms of late effects, and choose management techniques that minimize late effects and the consequent disturbance of everyday life [18].

The concept of self-management (SM) encompasses such abilities; one definition states SM in children and adolescents as “an active, daily and flexible process in which youth and their parents share responsibility and decision-making for achieving control of their condition, health, and wellbeing through a wide range of activities and skills. The goal of this increased responsibility is to develop the skills needed for the transition to adulthood and independent living” [19 , p. 92]. In a study interviewing CCS parents, the participants underscored that helping adolescents become their own advocates was an important part of their self-management support [20]. Generally, there is not much knowledge on the reintegration processes of CCS parents into daily life directly after the end of the treatment. A recent German study [21] found that even though most families successfully (re)adjust to a new normality, (re)integrating into daily life after cancer treatment may take a long time and remains difficult for both the child and the parents. On a positive note, some parents also described positive family changes such as improved communication with their child, increased trust, and a strengthened bond, indicating improved self-management skills. The fundamental core of SM is the active participation of patients in their own care [18]. This includes acquiring knowledge about the disease and treatment, engaging in shared decision-making with health care providers, and developing everyday coping skills such as self-monitoring and problem solving [11, 22]. To address CCS’s distinctive SM needs, it, therefore, seems crucial to offer additional multidisciplinary and tailored follow-up care to allow for ample opportunities to assess and teach SM skills.

Late childhood and teenage years are a period marked by significant changes, including role changes, shifting expectations, and new and challenging social interactions [23]. After completing cancer treatment, many children and adolescents wish to return to their school and other activities that their peers are engaged in [24, 25]. Such activities provide a sense of normality, and many report positive experiences when returning to school full-time and being among their schoolmates and friends on a more regular basis again [20]. However, cancer-induced physical and mental changes can contribute to feelings of being different from peers, even a long time after the completed cancer treatment [24, 25].

There is limited evidence on self-management in CCS [26]. Several frameworks have been developed to map self-management strategies in chronic disease, including cancer [27, 28]; however, the impact on CCS self-management strategies is scarce. Some evidence suggests that SM strategies may benefit adolescents in two different transitions: one from active cancer treatment to survivorship [29, 30]. Additionally, adolescent cancer survivors face another challenging transition, as they naturally transition to young adulthood; they also transition to the adult-focused health care system. This last transition is often described as even more demanding, as the adult health care system often is organized differently than the paediatric ward, expecting young cancer survivors to manage health care problems themselves without the parents’ involvement [31]. Therefore, including a focus on self-management skills in follow-up care would help prepare children and adolescent cancer survivors to take responsibility for their health before transitioning from paediatric to adult health care [30].

In Norway, as in many other countries, physical rehabilitation and SM techniques are currently not part of CCS routine follow-up care. We lack information about how SM skills can promote young CCSs’ re-entry to, and mastery of, everyday life after treatment completion. Given the rapid increase in the CCS population, there is an urgent need to improve our understanding of both the challenges young survivors face and how to best support them in managing these. To do so, we need improved knowledge of what challenges, needs, and resources they experience, seen from their perspectives. Therefore, we aimed to provide an in-depth description of the challenges faced by young CCS and the different approaches they use to manage everyday life. For future interventions to be effective, we need a detailed understanding of the features that influence the CCS self-management experiences and this study aims to contribute to building such knowledge.

## Methods

### Study design

This descriptive qualitative study is part of a large international multi-method and multi-centre project, “Physical Activity and Fitness among Childhood Cancer Survivors” (PACCS), aiming to determine physical activity levels and physical fitness of young survivors of childhood cancers, including perceived barriers and facilitators to being active, using a range of methods across three sub-studies. In the Norwegian qualitative sub-study, health care personnel, parents, and children and adolescents were interviewed about perceived barriers and facilitators of physical activity after cancer. This study reports on data from the individual interviews with the children and adolescents, focusing on themes related to the challenges they face and their self-management strategies.

### Participants

In total, 22 children and adolescents from the age of nine to 18 years (called young survivors) participated in this qualitative study; all were at least 1-year off-treatment and in remission. They were initially recruited to the first PACCS sub-study, which objectively measured physical activity levels, and were asked if they would be interested in participating in the subsequent interview study. Participants for the interview study were then purposively sampled from the first sub-study [31] to ensure variation in key factors affecting physical activity such as age, age at diagnosis, diagnosis, fatigue level, and place of residence. They were then contacted by study nurses to confirm participation and arrange a time and date for the interview. All but one of the survivors invited agreed to participate. Exclusion criteria were lack of consent and language difficulties.

### Semi-structured interviews

The interviews took place in connection with a medical follow-up consultation at the paediatric outpatient clinic at two university hospitals in Norway (Oslo and Bergen), both participating in the PACCS study. The hospitals combined serve approximately 75% of the Norwegian CCS population. The interviews followed a semi-structured interview guide [32] related to physical activity in daily living. Nineteen interviews were conducted at Oslo University Hospital by two female researchers (EHL and HCL) both experienced in paediatric cancer survivorship research. The three interviews at Haukeland University Hospital in Bergen were conducted by an experienced female nurse (SH). All interviews were audio-recorded and transcribed *ad verbatim*.

### Data analysis

**Fig. 1:**
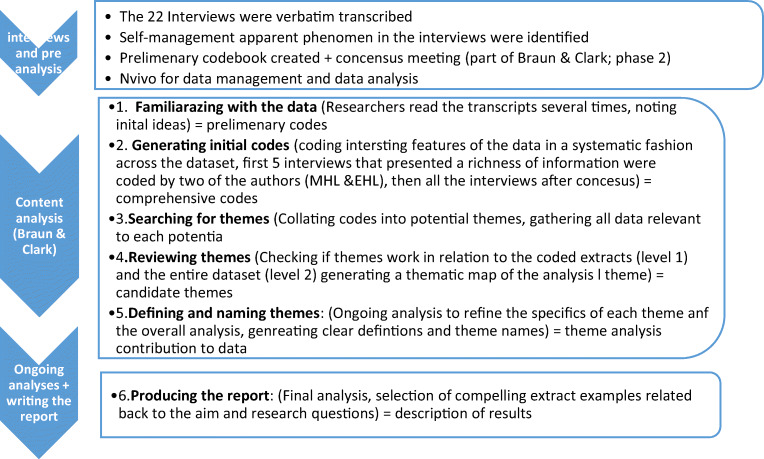
Overview of the analyzing process

The original aim of this sub-study was to explore young survivors’ experiences of engaging in physical activity in the years after treatment. Thus, the study did not set out to examine self-management; rather, it emerged as a significant phenomenon in the initial reading of the interviews. The authors, therefore, decided to analyze the data with a focus on perceived challenges and self-management strategies based on principles of thematic analysis as described by Braun and Clark [33]. We identified relevant features within all interviews generating codes using a hybrid inductive-deductive approach [34]. The first and second authors (MHL, EHL) did all the data coding, discussing any issues or disagreements (with HCL) to reach an agreement. The first codes were determined based on relevant self-management literature [18, 35, 36], and the data were coded deductively using the SM aware literature and theory. The material was then coded inductively to allow the participants’ voices to guide the analysis, enabling codes not relating to existing literature and theory to emerge. The thematic analysis was performed using the method by Braun and Clark’s six phases (i.e. familiarizing oneself with the data, generating initial codes, searching for the themes, reviewing the themes, defining and naming the themes, producing the report) [33] (see Fig. 1 for details of the process). All authors helped reviewing and refining the themes for clarity and completeness before the three overarching themes were finalized. Then, the themes were written using key quotes to support the presentation. NVivo qualitative data analysis software (version 12, 2018) was used for data management [37]. The study adhered to the consolidated criteria for reporting qualitative research (COREQ) guidelines [38].

### Ethics

Ethical approval was applied through the Regional Committee for Medicine and Health Research Ethics (REK-sør, 2016/953). All participants received age-appropriate information about the study aims. Informed written consent was provided by the parents of children < 18 years due to a request from the Ethics committee. Children < 16 years gave oral assent, while participants > 16 years provided written consent as they are considered to be of legal age in relation to health service rights and should therefore provide written consent similar to adults [39]. Participants were given the right to withdraw from the study at any time.

## Results

### Participants

A total of 22 semi-structured interviews were conducted, with eight female and fourteen male CCS. The participants had a mean age of 14.1 years (SD 2.7), and approximately 60% lived in a rural setting. The median time since treatment completion was 4 years (range: 1–12 years). There were eight different cancer diagnoses represented, with acute lymphoblastic leukaemia (ALL) as the largest group (36.4%) (Table 1).

**Table 1:** Participant description (N = 22)

Variables		N (%), mean (SD), median (range)
Sex	Female	8 (36.4%)
Mean age at interview		14.1 (SD 2.7)
Age groups	10–14 years	11 (50%)
	15–18 years	11 (50%)
Diagnosis	ALL	8 (36.4%)
	HL	3 (13.6%)
	NHL	5 (22.7%)
	CNS tumour	3 (13.6%)
	Solid tumours*	3 (13.6%)
Treatment	Chemotherapy	22 (100%)
	Radiation/brachytherapy	8 (36.4%)
	Bone marrow transplant	4 (18.2%)
Age at diagnosis (years)		Median 8 (0.5–15)
Years since treatment		Median 4.0 (1–12)
Habitation	Rural	9 (40.9%)
	Urban	13 (59.1%)

*ALL* acute lymphoblastic leukaemia, *HL* Hodgkin’s lymphoma, *NHL* non-Hodgkin’s lymphoma, *BT* brain tumours

*Outside CNS

### Results from the thematic analysis

Three main themes with eight sub-themes were identified through the thematic analysis [33], describing aspects of self-management related to the current daily life of CCS. The three themes are (1) *Managing everyday life with fatigue*, (2) *Building self-management competence*, and (3) *Cancer survivor as part of identity* (Table 2). See Table 3 for supporting quotes.

**Table 2:** Examples of codes and the sub-themes and themes emerged from the analysis

Themes	1. Managing everyday life with fatigue									2. Building self-management competence							3. Cancer survivor as part of identity?				
Sub-themes	Fatigue and its consequences			Pushing myself to master			Balancing activities			Mastering towards a new normal			Adjusting expectations and justifying choices			Changing arenas	Being open about the cancer experience		Positive benefits of cancer		
**Codes (examples)**	Beyond tired	Listening to the body’s signal	Use of different resources	Stepwise strategies	Expectations to own strength	Pushing boundaries	Recharging/timeout	Tough priorities	Behaviour adjustment	Concrete goals	Motivation	Changing arenas	New roles	New roles	Emotional adjustment	Done with the cancer experience Cancer means attention Positive consequences from cancer	Description of own resources Leaving it in the past	Accepting and go on Focus on present and future	Loss of affiliation	Better version of my self	Posttraumatic growth

**Table 3:** Illustrative quotes

Quotations in text	Illustrative quote
3.3. Managing everyday life with fatigue	
3.3.1.Fatigue and its consequences	
Q1	Female—16 years: “The others were tired, but I was beyond tired, I became so clumsy”
Q2	Female—17 years: “I’m very fatigued, I pushed myself too much for too long, and therefore I struggle a lot now, being tired”. (When asked when it started, she continues:) “I started noticing it right after I finished the treatment really, but I was confused, because when I had finished the treatment, then I felt that I should be healthy, so there should be nothing more wrong with me. When I finally told the doctor that I was very tired, it was ‘we know’ right away, that it was normal, but there was nothing they…., as I can recall that they talked about during treatment [Exposed breath]”
Q3	Male—15 years: “I still have some days where I have to take time off from school. I have to stay home and sleep all day, to take me up a bit - because then I’ve spent a lot of energy over time ...... Eeh and then the body simply takes over and says; ‘That’s enough, now you need to relax’ - so then I have to stay home and sleep”.
3.3.2. Pushing myself to master	
Q4	Male—15 years: “I like to push myself [laughs], I just think it’s good, and I think it’s good for the body and…. It is, after all, to be in shape, and, there is nothing worse than not being in shape somehow. And I can perform better on, really, everything……. I feel like when you get physically fit and can push yourself, it also becomes easier in school, in a way”.
Q5	Female—18 years: “I think I’m going to take the extra course next year, as it gives me general study privileges. And then my main goal is to become a nurse - but we’ll see if I can manage……, because it is a demanding career, you have to be “on the move” a lot, and that is difficult being as tired as I am all the time…”
3.3.3. Balancing activities	
Q6	Female—16 years: “After cancer treatment, I experienced having to be better at portioning out activities and stuff I do with my friends that require something of me. I have to make sure to retreat a bit, so I can participate, instead of having to lie straight out the next day and not being able to do anything. ........ it is difficult when fun things are happening, but, yes, it becomes part of everyday life, so you learn to do it, but it is not always easy”.
Q7	Female—17 years: “and then we had to borrow a wheelchair that we had at home. Then it was like.. if we went for a walk, I walked and Dad pushed the wheelchair, and then I went as far as I managed and then I sat down”.
3.4 Building self-management competence	
3.4.1. Mastering towards a new normal	
Q8	Female, 17 years: “To function in everyday life is most important, - that you manage to go for a walk, be able to be in school alone, without having to rely on a wheelchair, that you manage to lift your books yourself. Just being able to comb your hair and brush your teeth yourself, it was like getting lactic acid deluxe! – Just that – one day be able to do it, it’s a really great feeling of mastery!”
Q9	Male, 16 years: “I have these goals, after all. So now, I have started to run a little, because we have a hill test at school and I broke the record earlier this year (laughs). It has been standing for nine years, or something…”
3.4.2. Adjusting expectations and justifying choices	
Q10	Female, 18 years: “I have fatigue, so that’s why it is wise not to participate so much at middle school (years 8-10) as high school counts for much more. It’s better that I concentrate on that.”
Q11	Male 11 years: “Without the cancer I would have been higher, stronger and fitter, even stronger than my brother; Now I am like the smallest boy in my class”
Q12	Female, 13 years: “Listen to your body and do what you can, find something you can manage and maybe even love. If you feel ready for it, don’t be afraid to start again - because I was really scared of it - but I started, and am happy about it (laughs) - yes you will probably never be as good as you were, but you can still be very good”.
3.4.3. Changing arenas	
Q13	Male, 9 years: “I don’t really like football…. Yeah, I’m not very fast, and I am not that strong to kick the ball, as I used to…..when I somehow throw ... when I somehow can’t throw that far or throw so strong or somehow run so fast”. Interviewer: Why do you think that is? Boy: Well, after all, my body used all its forces to fight the cancer.
Q14	Female 12 years: “Football started to get a little boring so I wanted to try something new ..... I wanted to try gymnastics, but then I was not strong enough because you had to stay on a pole and then you had to pull your legs up. If you managed that, then you could start ... then after that, I started with kickboxing”.
3.5 Cancer survivor as part of identity?	
3.5.1. Positive benefits of cancer	
Q15	Female, 18 years: “Without the leukemia, much had been different. I would not have been so sure, what I want to do for a living, working as a nurse on this ward (pediatric oncology). I have a completely different view of what cancer is, not being as scared of it now as I used to be because I have seen that it can work out well sometimes”.
Q16	Female, 17 years: “I will not sit back and think damn, that the cancer was only shit, it really wasn’t - many nice things also happened, it was good times in a way (laughs)”. If I had not become sick, I would not have been the person I am today, and I am very satisfied being me”.
3.5.2. Being open about the cancer experience	
Q17	Female—17 years: “I have accepted that the cancer happened, now I want to go on, think ahead and not look back”. p. 153
Q18	Female, 16 years: “I’m a little addicted to being able to talk about it (the cancer disease) with someone because it was traumatic -because I went through something big”
Q19	Male, 15 years: “Hey, have YOU had cancer? Wow! - then you could have died.”

### Managing everyday life with fatigue

In most interviews, a recurring subject was fatigue’s impact on everyday life and functioning for the survivors. Many described how the fatigue controlled their decisions regarding activities and participation, and affected their management strategies. The fatigue also increased their need for support and necessitated explicit self-management skills, such as prioritizing some activities and forsaking others. The theme encompasses three sub-themes: (1) fatigue and its consequences, (2) pushing myself to master, and (3) balancing activities.

#### Fatigue and its consequences

During the interviews, almost all the young survivors shared clear descriptions of fatigue or fatigue-like symptoms and how it influenced their everyday lives. They generally described the fatigue using metaphors related to low energy: “I lost my power”, “I do not ever achieve a full charged battery”, and “When tired, I short-circuit”. The feelings of fatigue and reduced endurance were described as different from how they experienced being tired before the cancer treatment, and it was evident in the descriptions that the CCS were aware that their perceived lack of energy was different from that of peers (Table 3, 3.3, Q1).

Experiencing fatigue contrasted with several of the survivors’ expectations about the “after treatment period”, such as being even more active and feeling more vigorous than before getting cancer, as they now had become “really healthy”. Consequently, some had pushed themselves hard to meet these expectations but failed and experienced even more fatigue. Many were not given any information about fatigue during or after treatment. Thus, several survivors experienced feeling fatigued for a long time before mentioning it to health care personnel, who then told them that this was a typical late effect and to be expected. Some also concluded that having this information earlier would have made it easier to manage everyday life post-treatment (Table 3, 3.3, Q2). The survivors described several explicit self-management strategies on coping with fatigue, which they often had developed over time through trial and error. One of them was being aware of what “your body is telling you”, indicating that they had to be sensitive to and act on “fatigue signals” and adapt their activities accordingly to secure enough rest and to avoid “collapsing” afterward (Table 3, 3.3, Q3). They also described how fatigue and its disruptions to participation in activities made them more dependent on their friends and families’ practical support. It was primarily the parents that provided vital “fatigue-buffering” support by driving to activities and friends, waiting outside the school in case of acute tiredness, and organizing the family’s social life to prevent activity overload. Asking for, or accepting, support and help from friends and families was therefore described as an essential SM skill to buffer fatigue’s consequences on their everyday lives.

#### Pushing myself to master

A commonly described SM skill was to push themselves physically and mentally to master everyday activities limited by fatigue, including activities at school and with friends and family. Some of the older participants said that they believed that physically pushing themselves was a good thing, with positive spill-over effects to other parts of life, e.g. by giving more energy and a greater sense of mastery of their school work. They described this pushing as a kind of character-building quality and a self-management skill (Table 3, 3.3, Q4). However, for several of the participants, this strategy also had the down side of feeling like a struggle that ended in being even more fatigued. Several said that they were stubborn and conscientiously kept pushing themselves, even if they felt tired and knew they would experience fatigue afterward. Attending school was highly prioritized, which demanded a substantial amount of energy. To manage their academic aspirations, some survivors had developed step-by-step goals as a strategy to counteract their fatigue. For example, one girl treated for a brain tumour and suffering from severe fatigue focused only on few academic topics each year. Others had to rethink their academic aspirations; for example, one of the male participants chose to change school to become an electrician because he realized his academic abilities had changed post-treatment. Hence, the extent to which cancer and treatment had impacted everyday living was substantial, but somewhat unspoken. A female participant tells about her aspiration to become a nurse and her doubts about whether this will be possible due to fatigue (Table 3, 3.3, Q5).

#### Balancing activities

Many survivors talked about how balancing social activities and rest, recuperate and regain energy, was one of the most successful SM skills related to fatigue. Being with friends was highly prioritized, and the survivors were willing to sacrifice other things, such as school and physical activity, to be socially active. Because the sense of reduced endurance and a sense of weariness either occurred after some activity or were almost always present, they had to develop new skills to balance or conserve their energy to attend school and participate in activities with friends. They said that the balancing skills had developed over time and with many setbacks before achieving competence (Table 3, 3.3, Q6). Here, the parents also were important supporters; they supervised balancing activities and rest, for example, motivating re-engagement in sports, and providing time to rest and limit activities to prevent fatigue (Table 3, 3.3, Q7).

### Building self-management competence

It was evident that the young survivors developed new self-management skills and competencies by learning from their mistakes and re-calibrating their expectations about their capacity. At the same time, they developed concrete goals to achieve increased self-efficacy and use their resources for support, such as friends, siblings, and parents. This theme comprises three sub-themes: (1) mastering towards a new normal, (2) adjusting expectations and justifying choices, and (3) changing arenas.

#### Mastering towards a new normal

Being able to master everyday activities seemed to be an essential priority for the survivors following treatment completion (Table 3, 3.4, Q8). Setting concrete goals for achievements continued for many as an SM strategy to manage everyday life activities such as walking stairs alone, showering without assistance, or carrying their school books themselves. In these everyday tasks, the parents provided much practical support and enabled self-efficacy. The more active participants often had concrete physical goals and worked systematically and hard to regain their physical capacity. They were proud when talking about areas where they performed better than their peers or had reached the same physical level as before cancer (Table 3, 3.4, Q9).

#### Adjusting expectations and justifying choices

Some survivors struggled with persistently reduced physical capacity compared to their peers. This capacity gap was often understood as a consequence of the late effects of cancer or its treatment, such as limited lung capacity, reduced immune system, or fatigue, rather than something they had failed to achieve. Moreover, not participating at their peers’ level at school or in sports or social activities was explained by this gap. Reduced participation was also sometimes explained by the importance of prioritizing upcoming future academic tasks. For example, they reduced the number of school subjects in one school year to prepare for taking more important subjects the following year. As such, justifying their choices appeared to be a distinct SM strategy for mastering their lack of ability to measure up to their peers’ accomplishments (Table 3, 3.4, Q10). Another strategy to explain their reduced activity or capacity levels was exemplified by a 16-year-old girl struggling with post-cancer fatigue. She compared herself with her pre-cancer self, an energetic and bubbling 11-year-old. Explaining her changed behaviour and a much more sedate lifestyle to typical teenage developments helped her accept her current situation. Some children also talked about how acquiring knowledge that they were unlikely to become as fit or as strong as they had been before helped them adjust their expectations (Table 3, 3.4, Q11). Some then found new interests or hobbies they could master as an SM strategy (Table 3, 3.4, Q12).

#### Changing arenas

If the survivors had trouble measuring up to peers, an SM strategy was to change activities or social arenas. This was mostly related to sports and leisure, but several also talked about having changed to another school post-cancer. A typical change was choosing a school with fewer students and a more holistic view of academic learning than the traditional Norwegian public school system (Table 3, 3.4, Q13). In these arena shifts, the parents had a distinct role as facilitators and enablers to help ensure positive changes.

Especially when it came to team sports, running fast for an extended period, or running relay, the CCS had apparent difficulties performing at the expected level. Several quit structured activities such as football and basketball after the treatment as they felt they also let their team down by their poor physical performance. Experiencing reduced physical capacity or abilities made it sometimes challenging to re-enter old arenas and established roles with peers. Activities they used to enjoy were no longer fun because they felt it was challenging to master at anticipated levels (Table 3, 3.4, Q14).

### Cancer survivor as part of identity

As the CCS’s quote in the title of this paper illustrates, the cancer experience may integrate a new, and often different identity, and with it a realization that you have to do things differently post-cancer to master new tasks, or even re-master “old” tasks. Balancing between communicating the unique experience and still being able to be “normal” among friends seems like a challenging self-management strategy to master. This last theme has two sub-themes: (1) positive benefits of cancer and (2) being open about the cancer experience.

#### Positive benefits of cancer

Some survivors seemed to consciously or unconsciously engage in benefit finding as a SM strategy, hereby reporting a positive life change resulting from the struggle to cope with the cancer treatment and reframing the cancer experience as something with positive outcomes, making them a better version of themselves. This way of thinking seemed like a coping strategy that empowered the CCS and led to posttraumatic growth. They spoke of being more certain of what future occupations they wanted (Table 3, 3.5, Q15). Furthermore, several talked about how having had cancer had made them less afraid of it—because they had not only survived but, in some ways, emerged as a stronger and more competent person because of it (Table 3, 3.5, Q16).

#### Being open about the cancer experience

There was great variation among the participants regarding if, and if so, how they communicated their cancer experience in their day-by-day life. To regain a sense of normalcy, survivors selected to either be open about their history of cancer or await revealing this information to their peers, because they did not want to be “special” anymore. Some of the older survivors said they wanted to be honest about their illness, but many still did not tell their schoolmates anything unless directly asked. Several mentioned that only their closest friends knew about their cancer. Not talking about it could be seen as an SM strategy of “leaving it in the past” and, for some, to be more like “everyone else”. Several of the younger survivors said they do not think much about cancer themselves, and several of them said they felt “done” with the cancer experience and wanted to focus on the present and future (Table 3, 3.5, Q17). In contrast, several of the older participants seemed to actively use “talking about cancer” as an SM strategy to process what they had experienced. They described that the disease was a life-changing experience that they needed time to process and that repeatedly discussing it helped (Table 3, 3.5, Q18).

Some described that being a cancer survivor made them unique and made them stand out among their peers in a positive way. It seemed that they acknowledged and accepted having had cancer as a positive aspect of their new, improved identity. For these survivors, incorporating their cancer history as part of their identity made them feel “cool” and “different”, they sort of legitimized their cancer for both them and their peers. They expressed positive feelings when friends and schoolmates were “impressed” and somewhat shocked by their stories (Table 3, 3.5, Q19).

## Discussion

This paper aims to provide an in-depth description of the different approaches CCS use to self-manage everyday life, adding to currently limited knowledge of this particular age group of CCS and their SM skills. By identifying existing SM barriers and describing developed SM skills and an ongoing need for support, these results illustrate that CCS continues to have numerous challenges long after the cancer treatment has ended. These challenges are related to the ongoing physical and psychosocial effects of the cancer treatment, affecting their SM skills and quality of life both positively and negatively.

An essential result in the first theme was fatigue manifestation, and the survivors’ need to balance activity and rest to function both at school and home. The finding of severe fatigue as a common and debilitating late effect in CCS has been underscored in a recent Cochrane review [40]. Most of our study’s survivors described their fatigue as exhausting and strong, reducing physical function and social participation ability. Several were surprised by the fatigue. They had not expected any medical or psychological late effects from treatment, remaining a severe problem over time. These findings are in line with results in adult CCS [41].

The CCS described developing SM skills akin to energy economization and pushing themselves to manage their fatigue. They also talked about how they thought that physically pushing themselves was a positive SM strategy. In adult cancer survivors, exercise has a promising effect on cancer-related fatigue [42], possibly supporting exercise as a positive strategy also in younger ages. A systematic review of adult survivors’ interventions found that physical activity and psychological interventions improve chronic fatigue almost to the same extent [43]. Similar researches on CCS and comprehensive programmes for young survivors focusing on lifestyle and health behaviour are still lacking [44]. However, a novel report [45] from the International Late Effects of Childhood Cancer Guideline Harmonization Group found that physical activity interventions (e.g. aerobic and yoga) reduce fatigue in childhood, adolescent, and young adult cancers.

Some survivors said that they had received limited information about fatigue as a late effect. This may be due to the children’s young age at treatment completion; most of the late effect information was given to the parents. Also, health care professionals may be apprehensive about providing information about fatigue, being unsure of how to balance between providing sufficient information about late effects and creating unnecessary anxiety [46]. A quantitative study on adult CCS attending a late effect clinic found that very few survivors had knowledge of their specific treatment modes and reported a substantial underestimating of future health risks [47]. These findings are also supported in Norwegian studies of adult CCS, which conclude that although participants have a good recollection of treatment modalities, they had limited knowledge of the risk for treatment-related late effects [46, 48]. Another Norwegian study [49] analyzing video recordings of follow-up consultations with adolescent survivors found notable individual differences between the paediatric oncologists in information provisioning and a clear biomedical focus in the information provided. Furthermore, studies indicate that many adolescents are transferred to adult follow-up care without sufficient knowledge about their medical history or the risks for late effects [9, 50]. Thus, there is a need for further research on the effects of late effects, especially fatigue in the CCS group, and an increased focus on HCP’s communication of such late effects in all cancer trajectory phases. Self-management improves health outcomes by improving adherence to the follow-up plan and building the patient’s capacity to navigate challenges and solve problems [35, 51]. A recent systematic review concluded that survivors engaged in follow-up care have better health and educational outcomes, highlighting the need for lifelong follow-up and ongoing late effects education for survivors [52].

In the second theme, *building self-management competence*, it was apparent that to master everyday activities, the CCS had to modify expectations and previous behaviours both at school and socially. Studies have found that paediatric cancer sequelae may limit physical activity (PA) participation at school and in sports clubs, and survivors report frustration when not physically able to compete with their peers [53, 54]. As often told in our study, survivors may prefer using their energy in leisure and outdoor activities because those sports are generally less competitive than PA in sports clubs and at school. Hence, it seemed that it was not the physical activity itself they were trying to avoid, but more making a conscious avoidance of situations where they felt they did not satisfy the norm of peers.

The CCS talked about being similar to others and legitimized their ability gap with their history of cancer. This is in line with another qualitative study, where the adolescence and young adult (AYA) survivors distinguished themselves from others due to their cancer experience and their recent experience with associated consequences [55]. This need to feel different and, at the same time, for not being perceived as different by peers, is similar in the two studies. Hence, the CCS expresses an expectation to be treated as “normal”, while also expecting their unique history to be considered.

Many CCS talked about changing schools and other measures they took in order to reach academic goals. Previous research has found that Norwegian survivors of cancer diagnosed at a young age have an increased risk of being economically dependent and unemployed compared to healthy peers [56]. Furthermore, a recent meta-analysis revealed that the likelihood of unemployment among CCS is 50% greater than observed in the general population [57]. Hence, providing survivors with sufficient self-management support to successfully changing arenas and achieve coping in school and work settings seems of utter importance.

The last theme “Cancer survivor as part of identity” conveyed that several CCS described the cancer experience’s positive consequences. They concluded that the disease had made them better and stronger as individuals and given them the drive to make changes in approaching others and setting more ambitious future career plans. Such positive findings are also described in another interview study of adolescent survivors [58] where “finding meaning” surfaced throughout the interviews. Other studies with older survivors have described similar self-management strategies, such as drawing strengths from past experiences; acknowledging posttraumatic growth, self-awareness, and meaning-making; and strengthening family relationships [59, 60].

The various findings regarded strategies of being an “open” cancer survivor among peers, meant for that they could fulfil their need to repeatedly talk to friends about their experiences as part of their self-management strategies. Lepore and Revenson [61] propose that discussing cancer-related thoughts may enable one to process, react to, and cope with cancer. On the contrary, not thinking or talking about the cancer experience was communicated as helpful self-management strategies for some participants, wanting to move on with their lives. These latter findings are in concurrence with self-management strategies described by AYAS during cancer treatment, such as using distractions, waiting it out, staying positive, and maintaining a positive future perspective [62].

It was evident through the interviews that friends and, to an even greater extent, the families and especially the parents were essential sources of emotional, motivational, and practical social support. These findings are supported by another study of CCS [26] and show the importance of friends and family in CCS self-management. The paediatric self-management model by Modi et al. [63] underscores that for an approach to be effective, it is essential that in paediatrics, self-management means shared management between the youth and the parent(s) or caregiver(s). According to Modi et al., increased parental involvement and monitoring are associated with effective self-management. Thus, their participation seems promising to explore in future self-management support interventions.

### Study strengths and limitations

This study’s strength was the diversity of the sample, in terms of their age, gender, social context, time from completed treatment, experienced late effects, and place of residence covering many urban and rural regions of Norway. Furthermore, the interviewers’ extensive experience interacting with the study population may be considered a strength. Another strength was that the interviews were conducted at the hospital, a familiar place for the participants. They all chose to be interviewed without their parent present, indicating that they felt safe in this setting. The inclusion of young children is also a strength as very few studies include children from nine years. However, interviewing children as young as 9 years is multifaceted. Although the younger survivors were given an opportunity to voice their experiences and views, the older children’s interviews were generally more descriptive as their ability to reflect on consequences is more developed than the younger children. The fact that the participants were interviewed focusing on physical activity may be considered a study limitation; however, the material was rich and showed the CCS ability to reflect on more than facilitators and activity barriers.

### Clinical implications

Our research indicates that CCS possesses a range of desires, abilities, and skills to self-manage their health. Conceptualizing these skills within follow-up care may contribute to a feeling of mastery and increased satisfaction among CCS and may positively influence transitional processes to assist them in moving successfully to adult health care systems. An important priority is to help future CCS live healthy lives, increase their self-efficacy, find effective self-management strategies to minimize late effects from treatment, cope with ongoing psychosocial issues, and navigate follow-up care to optimize their long-term quality of life.

## Conclusion

The CCS expresses having gone through a life-changing ordeal that has made physical, psychological, and social imprints on their current life situation. Even years after the treatment, the CCS uses much effort to be normal compared to peers, and they have conscious self-management strategies to master everyday life. Nevertheless, the cancer experience is not dominating their current lives, and they are focused on current and future achievements and goals.
